# Selective antitumor activity of Tumor Treating Fields (TTFields) involving molecular factors in cancer cells and tumor microenvironment

**DOI:** 10.1016/j.tranon.2025.102556

**Published:** 2025-09-30

**Authors:** Ilaria Fuso Nerini, Rosy Amodeo, Maurizio D’Incalci, Monica Lupi

**Affiliations:** a Laboratory of Cancer Pharmacology, IRCCS Humanitas Research Hospital, Rozzano, Milan, Italy; b Department of Biomedical Sciences, Humanitas University, Pieve Emanuele, Milan, Italy

**Keywords:** Tumor Treating Fields (TTFields), Anticancer therapy, Preclinical studies, Solid tumors, Tumor microenvironment

## Abstract

The local application of low-intensity electric fields at intermediate frequencies (Tumor Treating Fields – TTFields) has emerged as an effective anticancer treatment in conjunction with chemotherapy and immunotherapy for several solid tumors. Despite this progress, the phenotypic and genetic determinants underlying tumor sensitivity to TTFields remain largely unexplored, representing a critical gap in our understanding.

This review provides a comprehensive analysis of preclinical and translational studies describing the cellular factors that influence the anticancer properties of TTFields. An overview of recent omics studies on the complex cellular and molecular processes initiated by TTFields has revealed important mechanisms of action that warrant further investigation for their therapeutic potential.

The goal is to identify effects that can be leveraged to develop rational, synergistic co-treatments with anticancer agents that have complementary modes of action. In particular, the ability of TTFields to modulate the tumor microenvironment and reverse the local and systemic immunosuppression could represent a promising strategy to enhance the efficacy of immunotherapy across different tumor types.

## Highlights

• Multiple factors are involved in tumor sensitivity and resistance to TTFields.
• Omics research has revealed molecular processes triggered by TTFields that inhibit tumor growth and modulate the microenvironment.
• The complexity of TTFields effects makes it challenging to identify predictive biomarkers.
• Co-treatment with TTFields and some anticancer drugs or radiotherapy show promise.
• TTFields’ ability to alter the tumor microenvironment and immune response is of therapeutic interest for novel co-treatments.

## Graphical abstract

**Figure:**
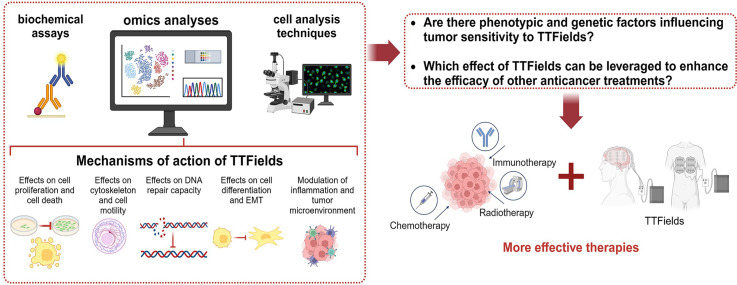

## Introduction

Despite significant advances in cancer therapy, important challenges remain, including therapy resistance, tumor heterogeneity, immune evasion, and systemic toxicity, all of which must be addressed to further improve the survival rate for this disease. Conventional pharmacological and immunological treatments are often limited by adverse effects and the tumor’s ability to adapt through mechanisms like genetic mutations, metabolic flexibility, and remodeling of the tumor microenvironment (TME). These limitations underscore the urgent need for more effective and tolerable therapeutic strategies that go beyond traditional approaches.

In this context, Tumor Treating Fields (TTFields) emerge as a promising non-invasive modality that applies low-intensity, intermediate-frequency, alternating electric fields to target cancer cells. TTFields therapy holds great potential across multiple cancer types, as their efficacy is accompanied with a highly tolerable safety profile [[1], [2], [3], [4], [5], [6]]. Their use has been introduced into clinical practice for the therapy of glioblastoma (GBM), pleural mesothelioma (PM), and non-small cell lung cancer (NSCLC) following the demonstration of a significantly improved overall survival compared with standard therapy alone [[5], [6], [7]]. Some patients receiving TTFields are long-term responders, suggesting that this therapy has the potential to significantly disrupt key features of cancer malignancy [[8], [9], [10]]. Nevertheless, clinical outcomes are not uniformly positive for all patients, indicating that further research is needed to understand and improve the efficacy of TTFields across different patient populations.

As with many anticancer therapies, identifying predictive factors enables the stratification of patients according to their expected sensitivity to specific treatments. This approach helps avoid unnecessary medication and increases the likelihood of positive outcomes, aligning with the prevailing concept of personalized medicine. Nevertheless, there is limited evidence regarding which genetic or phenotypic characteristics of tumors are associated with the response to TTFields. The discovery of these molecular drivers could provide crucial insights for new research aimed at enhancing their antitumor efficacy, either as monotherapy or with other physical, chemical or biological anticancer therapies.

Preclinical investigations are essential to this purpose. Thus far, limited studies have focused on characterizing the heterogeneity of tumor responses and identifying key factors that contribute to the increased mortality of tumor cells compared to normal tissues. Conversely, numerous studies using *in vitro* and *in vivo* models have revealed the complexity of TTFields effects on cancer cells, some of which have been recognized as mechanisms that drive their antitumor activity. The wide range of physical and biological effects exerted by TTFields may contribute to their significant antitumor efficacy [11], but also complicates the identification of factors influencing tumor sensitivity.

In this review, we provide a comprehensive overview of preclinical and translational studies that have attempted to elucidate the mechanisms of sensitivity to TTFields. The literature search was conducted in MEDLINE using “Tumor Treating Fields” and its synonyms as keywords, spanning from January 2004 to May 2025. Special emphasis is placed on studies that apply omics approaches (including targeted gene sequencing, microarray analysis, whole genome and transcriptome sequencing, kinomic or proteomic analyses) to propose novel strategies for antitumor treatment.

## Selective activity and predictors of responsiveness to TTFields

### Physical features

**Fig. 1:**
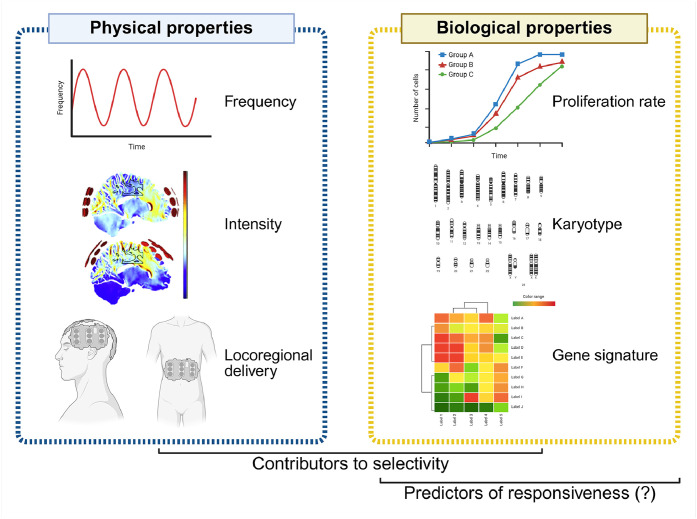
**Physical and biological properties involved in the selectivity of TTFields against cancer cells.** Frequency, intensity, and locoregional delivery, as well as cell proliferation rates, karyotypes and gene alterations have been examined for their role in mediating TTFields selectivity. However, few study were aimed at determining predictors of responsiveness to this treatment. Created with BioRender.com.

Certain physical properties of locally-delivered TTFields, such as frequency and intensity, enable the effective targeting of cancer cells while minimizing damage to normal tissues (Fig. 1).

TTFields frequency is currently empirically optimized during the preclinical phase of therapy development, based on the tumor type to be treated. In vitro experiments have shown that maximal cytotoxicity of different tumors occurs at different frequencies [[12], [13], [14], [15], [16], [17], [18]]. This observation has been translated into clinical trials and clinical practice guidelines: TTFields at 200 kHz are applied in the treatment of patients with GBM or ovarian cancer, while 150 kHz are used against PM, NSCLC, pancreatic cancer, gastric cancer, hepatocellular carcinoma, and brain metastases from NSCLC [[19], [20], [21]].

The intensity is another physical parameter that strongly correlates with cytotoxic and cytostatic effects of TTFields, as demonstrated by several *in vitro* studies [17,[22], [23], [24], [25]]. TTFields are delivered at 1 to 3 V/cm root-mean-square (RMS) in animal models [26,27], as well as in patients. The actual electric field intensities reaching tumor cells are influenced by different factors, primarily the distance from the electrodes and the dielectric characteristics of the crossed tissues [28]. Mathematical modelling has attempted to assess how the intensity delivered to the target varies in relation to these parameters. Blatt et al. demonstrated that electric field intensities delivered at the abdominal area of healthy rats decreases by about 15–40 % from a depth of 1 to 3 cm, reaching an intensity at least 10 time lower than the effective one in organs distant from the area of application [26].

In addition to local delivery, the low conductivity of some normal tissues contributes to the favorable safety profile of TTFields. For instance, the high impedance of the bone and bone marrow protects these tissues from TTFields, as the intensity in these regions is 100 times lower than in the near environment [13].

### Individual biological parameters

**Fig. 2:**
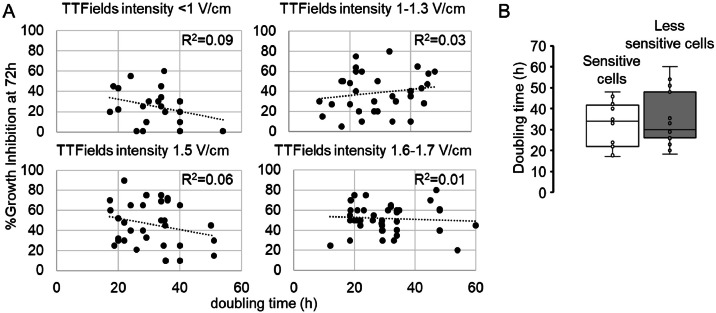
**Lack of correlation between cell doubling time at baseline and sensitivity to TTFields.** A) Growth inhibition values after 72 h of exposure to TTFields, calculated as the percentage of control cells, were collected from different preclinical studies involving several cancer cell lines (Supplementary Table). All data are plotted versus cell doubling time, after grouping the cells based on the treatment intensity. No significant correlation between the two parameters is observed. B) After dividing the cells into two populations with different sensitivity to TTFields, doubling time distributions were compared. Doubling times are not statistically different in the two populations according to Student’s t-test *p* > 0.05.

Besides the physical parameters characterizing TTFields treatment, some studies have investigated the role of biological features in the response of different cancer cell lines to TTFields (Fig. 1). To explore this further, we conducted a comprehensive analysis of the preclinical data available in the literature (Supplementary Table). We retrieved 39 publications detailing the results of *in vitro* experiments analyzing the effects of TTFields, and we identified two cell populations based on their responses after 72 h of treatment: i) sensitive cell lines, which exhibit more than 50 % growth inhibition at TTFields intensity below 1.2 V/cm RSM (A375SM, BxPC-3, CD473, CD484, HepG2, JIMT-1, LN-18, MSTO-211H, NCI-H460, U251-MG, U-373 MG), and ii) less sensitive cell lines, which show less than 50 % growth inhibition when treated at 1.7 V/cm RSM (AsPC-1, DAOY, HCC38, HCC827, LN-229, MCF-7, MDA-MB-231, NCI-H1299, NCI-H520, SF188, U-118 MG, U-87 MG). In our investigation of potential associations between sensitivity to TTFields and various biological characteristics, we began by examining cell proliferation rates. This parameter was initially hypothesized to be a key factor in TTFields activity, based on the rationale that low-proliferating cells are less sensitive to a treatment that primarily affects the mitotic spindle assembly [[29], [30], [31]]. However, among the two identified cell populations, we did not find any correlation between the sensitivity to TTFields and doubling time, which reflects the cell growth rate at baseline (Fig. 2). Considering the complexity of TTFields-induced effect, this result is not entirely unexpected. The antimitotic property of TTFields are likely to have a greater impact on highly proliferating cells. However, a range of transcriptional modifications – affecting not only genes involved in cell proliferation and cell cycle progression but also those related to cell death, as well as protein and RNA metabolism – can occur in low-proliferating cells [32], explaining their sensitivity to this treatment. Further research is warranted to confirm the effectiveness of TTFields in this specific context, as even patients with slowly proliferating tumors could potentially benefit from this therapy.

**Fig. 3:**
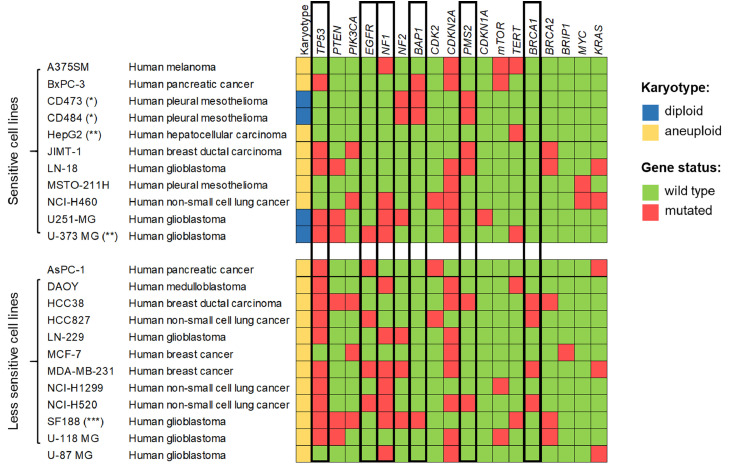
**Karyotype and mutational status of certain genes in sensitive and less sensitive cell lines to TTFields treatment.** Some studies have hypothesized that these genes may play a role in the response to TTFields (Table 1). Sensitive cell lines experience a growth inhibition greater than 50 % after 72 h of treatment with TTFields at an intensity of <1.2 V/cm RSM, while less sensitive cell lines experience a growth inhibition less than 50 % when treated with an intensity of 1.7 V/cm RSM. Karyotype and mutational status for each cell line were derived from the Cell Line Project of COSMIC database (https://cancer.sanger.ac.uk/cell_lines), unless specified otherwise. (*) Karyotype and mutational status derived from [24]. (**) Karyotype and mutational status derived from www.cellosaurus.org. (***) Karyotype and mutational status derived from [92].

In the search for characteristics that confer sensitivity to TTFields, karyotype is another parameter that has been investigated (Fig. 3). By comparing the results from various published studies, we found that a higher number of diploid cells was observed among the 11 cell lines identified as sensitive, while the less sensitive subgroup included only aneuploid cells. From this observation, it can be inferred that karyotype may influence treatment sensitivity. However, the available data are insufficient to draw definitive conclusions regarding the relationship between chromosomal composition and therapeutic response. In an ad hoc study using ten cell lines, Giladi et al. claimed that any clear correlation was present between chromosomal number and clonogenic survival following TTFields treatment [31].

The use of contemporary genomic next-generation sequencing (NGS) techniques has allowed detailed investigation of molecular alterations associated with the response to TTFields. Variations in structure or levels of specific genes and their related proteins that could confer sensitivity to TTFields have been analyzed (Table 1). So far, only a few studies have investigated the impact of the mutational status of a particular gene on TTFields response, using the same cell line engineered to express either the wild type or the mutant form of the gene of interest [33,34]. Generally, comparison among different cell lines have been considered to address this issue. However, it cannot be excluded that the genetic background of the models used may influence the role of a specific gene during and after TTFields exposure. By dissecting the results of the *in vitro* experiments included in our inter-study analysis, we aimed to identify correlations between treatment sensitivity and the status of specific genes (Fig. 3). The less sensitive cell population is characterized by a higher percentage of cells harboring mutations in *TP53, EGFR, NF1* and *BRCA1*, along with a lower proportion of mutations in *BAP1* and *PMS2*, compared with the sensitive group.

**Table 1:** **List of studies integrating data on molecular factors affecting the response to TTFields and the factors influenced by TTFields treatment.** Downregulated pathways are indicated in red, while upregulated pathways are shown in green. In studies analyzing patient biopsies we indicated the number of samples (TTFields-treated / total). (These references cited in this Table [[24], [30], [35], [36], [37], [38], [39], [40], [41], [43], [44], [46], [47], [72], [75], [79], [93], [94]]).

The lack of a clear correlation between specific gene mutations and TTFields sensitivity is likely multifactorial. Contributing factors may include the limited number of studies addressing this aspect, the small number of cell lines analyzed, and the heterogeneous genetic backgrounds of the models used, including other cellular parameters that may influence their response to TTFields. Nevertheless, the observed trend of gene enrichment in the less sensitive population could serve as a valuable starting point for future investigations.

To avoid a potential misinterpretation of these results, two types of investigations could provide insight into the genetic profiles that contribute to sensitivity to TTFields: i) using mutant cell lines genetically altered with specific molecular changes, and ii) examining cell lines that either exhibit innate resistance to TTFields or have been repeatedly exposed to them [35]. Comparing the effects induced by TTFields in these cell lines and their isogenic counterparts through multi-omics analyses could help identify biological characteristics associated with sensitivity or resistance to this treatment.

### Integrated biological parameters and insight from omics data

To date, few studies, exclusively focused on GBM, have examined the comprehensive molecular profile of tumor biopsies in relation to their response to TTFields [30,[35], [36], [37], [38], [39]]. The details of these clinical studies, along with the preclinical ones, are presented in Table 1.

Three of them, using a targeted NGS approach, have indicated that specific gene mutations in GBM are associated with better outcomes following TTFields therapy. These include driver alterations in *NF1*, wild-type *PIK3CA* and *EGFR* [36], mutated *PTEN* [37], as well as *MGMT* promoter methylation and *TERT* mutations [39]. Tumors harboring *TP53* or *EGFR* alterations were associated with a reduced progression free survival [39]. However, a more recent study by She et al. found no statistically significant correlation between patient survival and mutations in a panel of 16 genes, including those previously identified as critical [38]. The challenging interpretation of the results arises from several factors: the limited number of studies, the small study population, the concurrent use of standard chemotherapy, the use of a targeted approach rather than a whole genome analysis, and the fact that post-treatment biopsies are only available upon recurrence, typically in patients with poor response. Additional limitations of two studies include the involvement of patients with recurrent tumors, where mutational status was evaluated based on the initial resection without considering the evolution of genetic alterations [37,38]. Moreover, all these analyses were retrospective, which introduced potential bias in patient selection.

The results of other NGS analyses focusing on the immune response and the modulation of the TME by TTFields were instead more consistent. The expression levels of 712 immune-related genes were analyzed in tumor tissues from 12 newly diagnosed GBM patients, both before and after treatment with TTFields plus chemoradiation [30]. It was observed that TTFields modulated the transcription of 31 immune-related genes, resulting in a notable shift from a pro-tumoral to an anti-tumoral immune signature. Furthermore, single-cell and bulk RNA sequencing of peripheral blood mononuclear cells (PBMC) collected from patients with newly diagnosed GBM, before and after treatment with TTFields and temozolomide, revealed that therapy promotes a strong activation of adaptive immunity through a type 1 interferon-based (T1IFN) mechanism. Additionally, a gene panel signature indicative of TTFields influence on T cell activation and clonal expansion was identified [40].

Preclinical studies have applied omics techniques in both *in vitro* and *in vivo* models, primarily to explore the effects of TTFields. These investigations have contributed to a complex understanding by revealing a broad range of treatment-induced alterations. However, they have faced challenges in identifying predictive biomarkers useful for patient stratification and in pinpointing specific pathways to effectively guide future clinical trials. Using cell models of mesothelioma and NSCLC, two independent studies hypothesized that the efficacy of TTFields could potentially depend on the cells’ capacity to repair DNA damage [24,41]. By harnessing the potential of single-cell RNA sequencing and machine learning methods, Chen et al. identified a glioma stem cell (GSC) signature that may confer a particular sensitivity to TTFields [42]. This signature comprises 11 genes encoding proteins with significant roles in stem cell maintenance and growth (*ANXA1*), cell differentiation (*IGFBP2*), cell migration (*POSTN, SOX8*) and angiogenesis (*SCG3, TNFRSF12A*). Consistent with this, a recent study using GBM cell lines and primary cultures derived from GSCs, made resistant to TTFields after prolonged exposure, found that this resistance was associated to the overexpression of the prostaglandin E2 receptor 3 and zinc finger 488 (PTGER3-ZNF488) axis [35]. The expression of these genes was also monitored across eight cell lines of various human and murine cancers (bladder epithelial carcinoma, triple negative breast carcinoma, cervical carcinoma, pancreatic ductal adenocarcinoma, lung adenocarcinoma, renal cell carcinoma, and melanoma), and PTGER3 and ZNF488 were found to be upregulated after 24 h of TTFields exposure. The upregulation of this pathway, which promotes the self-renewal of GSCs, was further validated using clinical specimens from patients with recurrent GBM. These findings support the notion that this axis is involved in the mechanism of TTFields resistance and represents a potential marker of sensitivity to treatment. However, further translational and clinical studies, involving a larger patient cohort, should be performed to definitively confirm that the PTGER-ZNF488 axis is essential for TTFields resistance and to validate it as a predictive marker for sensitivity to the treatment across different tumor types. The therapeutic implications of these findings could also be significant, as pharmacological inhibition of this axis might prevent TTFields resistance and enhance the efficacy of simultaneous exposure to TTFields and other cytotoxic therapies.

## Suggestions for new rational therapeutic strategies

**Fig. 4:**
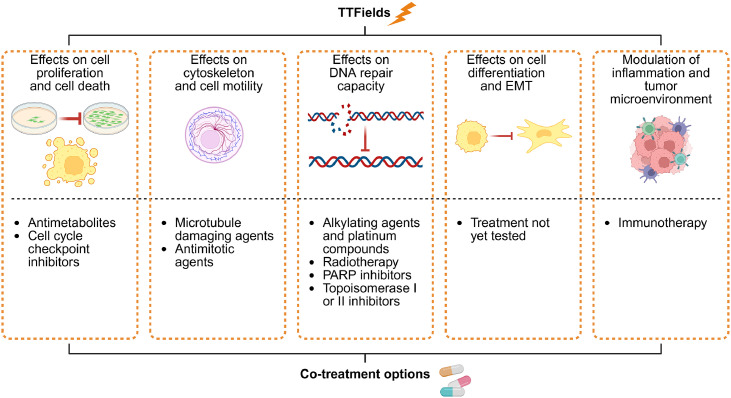
**Main mechanisms of action of TTFields derived from omics studies that could be exploited to design co-treatment options.** Some of these co-treatments have already been tested in preclinical or clinical studies, while others, such as those involving agents that affect cell differentiation and epithelial to mesenchymal transition (EMT), represent potential therapeutic strategies that are worthy of investigation. Created with BioRender.com.

The results of the integrated and omics molecular analyses have enabled the identification of five main pathways modulated by TTFields activity, suggesting potential strategies for designing synergistic co-treatment therapies with TTFields (Fig. 4). Preclinical studies have demonstrated that some anticancer agents produce significant antitumor effects when applied with TTFields, holding promise for future clinical application. In particular, a recent study, using a specific bioinformatics tool, identified the activation of similar pathways in cancer cells treated with TTFields or other drugs, including antimitotic agents, various DNA-damaging agents, antimetabolites, DNA methylation and chromatin modifiers, and DNA alkylating agents. This confirms the versatile profile of TTFields therapy, potentially allowing their application across various tumor types, without the toxicity often associated with systemic drugs [32].

### Effects on cell proliferation and cell death

The antiproliferative activity of TTFields was initially attributed to their direct disruption of mitosis [29]. However, recent omics analyses have revealed a more complex mechanism involving multiple cellular pathways. Kinomic studies [43] identified several kinases suppressed by TTFields, with a predominant focus on ERK activity. Furthermore, Xu et al., through transcriptional and proteomic sequencing, suggested that the lncRNA antisense to CDK2 (CDK2-AS1), which stabilizes CDK2 mRNA, is a target inhibited by TTFields. Knockdown of CDK2-AS1 was shown to promote cell cycle arrest and apoptosis in GBM cells exposed to TTFields [44].

A recent study have also explored how TTFields influence key signaling pathways such as PI3K/AKT in a panel of different cancer cell lines [45]. They demonstrated that AKT activity increases over time during TTFields exposure and is modulated by cell-surface and cell-cell interactions, with focal adhesion kinase (FAK) and N-cadherin playing roles in promoting AKT phosphorylation and activating cell survival pathways. Importantly, pharmacological inhibition of PI3K enhanced the antiproliferative effects of TTFields and augmented cell death, both *in vitro* and *in vivo*, indicating that suppression of this pathway can potentiate treatment efficacy.

The interplay between TTFields and cellular process such as autophagy and senescence [46] further complicates their mechanistic landscape, leaving the question of their role in these pathways open. Kim et al. [47] found that autophagy activation contributes to the activity of TTFields in GBM cells by decreasing AKT2 levels, mediated by the upregulation of miR-29b This suggests that autophagy may function as a survival mechanism during TTFields treatment. Conversely, Xu et al. [44], aligning with findings by Shteingauz et al. [48], argued that inhibiting autophagy could enhance TTFields efficacy, implying that its upregulation might serve as a survival mechanism during treatment.

Building on these mechanistic insights, researchers have evaluated TTFields in preclinical settings alongside various anticancer agents that target cell proliferation, including conventional chemotherapeutics and targeted kinase inhibitors. The addition of TTFields to conventional chemotherapeutics, such as antimetabolites (e.g., 5-fluorouracil [15,49,50], pemetrexed [14,17], gemcitabine [15]) has demonstrated enhanced antitumor activity, exhibiting synergistic or additive effects across different tumor models. Notably, a synergistic reduction in clonogenic survival was observed in GBM cells treated with TTFields and drugs targeting G2M checkpoint, such as Wee1, Chk1 or ATR inhibitors. Disruption of TTFields-induced G2M arrest impairs DNA damage repair mechanisms, thereby increasing cytotoxicity and improving overall treatment efficacy [51,52].

### Effects on the cytoskeleton, mitotic spindle and cell migration

Effects on the cytoskeleton were among the first recognized for TTFields [29]. The influence exerted by the electric field on polar molecules hampers the formation of the mitotic spindle, leading to impaired abnormal chromosome segregation [31]. In recent years, additional details have emerged that contribute to a more precise understanding of the interaction between TTFields and the cytoskeleton, suggesting a role of TTFields in limiting cancer cell motility. Indeed, Voloshin et al. demonstrated that TTFields increase the number and size of focal adhesions and alter the structure of actin filaments via the GEF-H1/RhoA/ROCK signaling pathway [53].

The potential of TTFields to disrupt mitotic spindle formation has been effectively harnessed through innovative co-treatments. For example, Kessler et al. demonstrated the efficacy of a drug blocking the spindle assembly checkpoint (SAC) when administered with TTFields in two human GBM cell lines, resulting in enhanced mitotic catastrophe [54]. Similarly, Krex et al. noted a higher percentage of polyploid cells in primary cultures of newly diagnosed and recurrent GBM after co-treatment with TTFields and an Aurora B inhibitor compared to each treatment alone, leading to a significant increase in cytotoxicity [34].

Increased antitumor efficacy has been observed *in vitro* and *in vivo* models treated with TTFields and mitotic inhibitors, specifically taxanes [[14], [15], [16],^55^,^56^]. TTFields may even enhance paclitaxel sensitivity in resistant cells [14,22]. Disappointingly, the phase III ENGOT-ov50/GOG-3029/INNOVATE-3 study, which evaluated the efficacy and safety of co-treatment with TTFields and paclitaxel compared to the drug alone in patients with platinum-resistant ovarian cancer, did not demonstrate a significant improvement in overall survival for the subgroup receiving both agents [57]. At the same time, despite the favorable trend observed in the median overall survival of patients with metastatic NSCLC treated with TTFields and docetaxel in the phase III LUNAR study, the improvement was not statistically significant compared to the subgroup treated with the drug alone [6]. The results obtained during these clinical trials demonstrated that translating preclinical co-treatments to clinical settings is complex and requires further investigation to effectively enhance treatment efficacy. The discrepancy observed between preclinical and clinical outcomes may stem from multiple factors. One might speculate that the similar mechanisms of action of TTFields and taxanes require a finely controlled timing of administration to elicit the synergism observed in preclinical settings, conditions that cannot be easily reproduced in clinical setting [58]. Moreover, results from the ENGOT-ov50/GOG-3029/INNOVATE-3 study highlight the potential value of more rational, biomarker-driven patient stratification in improving clinical trial outcomes. In this specific case, post-hoc analysis suggested that patients with platinum-resistant ovarian cancer who had not previously received pegylated liposomal doxorubicin could benefit from the association of TTFields with paclitaxel, showing significant longer overall survival compared to paclitaxel alone (16.0 vs 11.7 months; p = 0.03) [57]. One possible explanation for this finding is that doxorubicin-induced tumor fibrosis and associated changes in tissue conductivity may affect the distribution of TTFields within the tumor, thereby reducing their therapeutic efficacy [59,60].

### Effects on DNA repair capacity

The first paper demonstrating the ability of TTFields to modulate DNA damage repair pathways was published in 2017 [41]. Using a panel of NSCLC cells with different sensitivities to TTFields, the authors reported a significant downregulation of the BRCA1-dependent DNA-damage response pathway in all cell lines. Notably, this modulation was more pronounced in the most sensitive cells. These findings were further validated in PM [17], ovarian cancer [61], and GBM cell lines [62]. Consistently, cells exposed to TTFields exhibited a higher incidence of DNA double-strand breaks and a lower expression of proteins involved in the Fanconi Anemia (FA)-BRCA DNA repair pathway.

The ability of TTFields to impair the DNA repair machinery has been therapeutically harnessed to enhance the antitumor effects of radiotherapy [41,63,64], as well as those of alkylating agents or platinum compounds (e.g., temozolomide [62,65], cisplatin [14,17,66]), and anthracyclines (e.g. doxorubicin [22,25,55,67]), yielding promising preclinical results. Preliminary studies on the feasibility and tolerability of co-treating patients with GBM using TTFields and radiotherapy indicate that this approach is viable and has minimal toxicity [68]. The addition of a third agent, such as a DNA-damaging agent or a PARP inhibitor, has been shown to further improve efficacy [66,67,[69], [70], [71]]. The ongoing phase III TRIDENT trial is currently evaluating the efficacy of the triple combination of TTFields, radiotherapy, and temozolomide in patients with newly diagnosed GBM (ClinicalTrials.gov Identifier: NCT04471844).

### Effects on cell differentiation and epithelial to mesenchymal transition (EMT)

Recent studies across various tumor types, suggest that part of the antitumor effect of TTFields may be related to the restoration of cell differentiation. Specifically, transcriptomic analyses performed on two human GSC lines exposed to TTFields revealed an enrichment of the NOTCH signaling pathway and a neuroglial development signature, along with astrocyte and oligodendrocyte cell differentiation. These findings imply that this treatment can induce a transition from a mesenchymal to a proneural phenotype [72]. This underscores the intricate role of NOTCH signaling in regulating a wide range of cellular and developmental processes. Although traditionally recognized as an oncogene due to its role in promoting neural stem cell activity, recent evidence showing improved prognosis in GBM patients with high expression of certain NOTCH target genes have prompted a reevaluation of its potential as a tumor suppressor [73]. While elucidating the precise role of NOTCH in tumor development is beyond the scope of this review, modulating this pathway could represent a promising therapeutic strategy to promote differentiation and improve outcomes for patients with GBM.

On the other side, a recent study has demonstrated that prolonged exposure of GBM cells to TTFields may upregulate genes associated with cellular stemness, leading to therapy resistance. Specifically, the overexpression of the *PTGER3-ZNF488* axis observed in resistant clones has been shown to enhance self-renewal capabilities. Furthermore, inhibiting PTGER3 in TTFields-sensitive GSCs prevents the development of resistance [35]. Co-treatment with a PTGER3 inhibitor or even with aspirin, which is a known inhibitor of the upstream enzyme cyclooxygenase 1/2 (COX1/2), could offer new therapeutic strategies to extend the efficacy of TTFields.

The impact of TTFields on cell differentiation and EMT was further validated in other tumors. Oh et al. showed that, in osteosarcoma cell lines, TTFields induced a downregulation of EMT markers such as vimentin, N-cadherin, TWIST, and SNAIL proteins, that are also responsible for cell migration and invasiveness [74]. Through an analysis of the transcriptional profile of PM in a murine model, Sarkari et al. observed a significant downregulation of the glycoprotein Tenascin C (TNC), known to be involved in the EMT transition, and the vascular endothelial growth factor (VEGF), a key player in cell adhesion, motility, and also in EMT [75].

### Modulation of inflammation and tumor microenvironment (TME)

The first evidence that TTFields could play a role in modulating the TME, particularly in T cell recruitment, was presented by Kirson et al. [22]. Using a rabbit model with intra-kidney cancer, they found that TTFields reduced the number of lung surface metastases and prolonged survival. These effects were associated with extensive peri‑ and intra-tumoral immune cell infiltration, indicative of a T-cell mediated response. The immune-activating role of TTFields was further confirmed in different tumor murine models [76,77], where increased circulating levels of proinflammatory high mobility group box 1 protein (HMGB1) and elevated intratumoral levels of phosphorylated eukaryotic translation initiation factor 2 (p-eIF2), which triggers calreticulin translocation to the cell surface of dying cells, were observed in TTFields-treated mice. Both of these alterations are indicative of immunogenic cell death (ICD) induction. Moreover, TTFields were shown to promote the maturation of dendritic cells *in vitro*, as well as the recruitment of immune cells *in vivo*. A possible link between TTFields and immune activation involves their capacity to interfere with mitosis, causing the release of cytosolic micronuclei that activate DNA sensors such as cyclic GMP-AMP synthase (cGAS) and absent in melanoma 2 (AIM2), along with their associated inflammasome pathway cGAS/stimulator of interferon genes (STING) and AIM2/caspase 1. This process triggers the production of proinflammatory cytokines, type 1 interferons (T1IFNs), and T1IFN-responsive genes. Single-cell sequencing analysis has revealed that immune checkpoints molecules – including programmed death ligand 1 (PD-L1), cytotoxic T-lymphocyte-associated protein 4 (CTLA-4), and T cell immunoglobulin and ITIM domain (TIGIT) – are upregulated on the surface of tumor cells following TTFields treatment, immediately suggesting that co-treatment with immune checkpoint inhibitors could provide significant benefits in this context [40].

Based on TTFields ability to elicit ICD and activate adaptive immunity, their co-application with other immunomodulating agents and immune checkpoint inhibitors have been investigated. Concomitant treatment with TTFields and immune checkpoint inhibitors (especially anti-PD-1) led to an increase in leukocyte infiltration into the tumor tissue, together with a concurrent reduction in immunosuppressive infiltrating macrophages and myeloid-derived suppressor cells [76,77]. TME modulation in NSCLC models was observed to be possible thanks to the activation of CCL2/8-CCR2 and CXCL9/10-CXCR3 axes, which could also be considered potential biomarkers. Their levels in peripheral blood could be assessed to monitor patient response to TTFields and anti-PD therapy in this tumor type [78].

The phase III clinical trial LUNAR demonstrated the benefits of the co-treatment with TTFields and anti-PD-1 in patients with advanced-stage NSCLC, compared to immune checkpoint inhibitor therapy alone [6]. However, the heterogeneity of this type of cancer and the evolution of the TME at different stages of the disease may influence the response to TTFields. Therefore, further studies on the molecular mechanisms of TTFields-induced antitumor immune response, as well as the selection of the optimal timing, duration, and intensity of TTFields therapy may help optimize this treatment regimen even further [79].

The interaction of TTFields with the stroma, particularly with mesenchymal stromal cells, represents a new interesting issue to address. It has been demonstrated that these cells play a crucial role in tumor development by increasing angiogenesis, promoting invasion and metastasis, and suppressing antitumor immune response [80]. Preliminary data demonstrate that TTFields exert multiple effects on mesenchymal stromal cells, including reduced proliferation, induction of apoptosis and senescence, and impaired migratory potential [81].

## Unsolved questions and future perspectives

Preclinical studies have demonstrated that TTFields elicit a broad spectrum of effects beyond mere physical disruption. In fact, the role of biological factors in determining therapeutic outcomes is becoming increasingly evident. TTFields can modulate various cellular processes, including cell proliferation, cell death, cytoskeletal dynamics, migration, DNA repair, differentiation, EMT, inflammation, and anticancer immunity. These diverse effects contribute to their selectivity and relatively low toxicity, allowing TTFields to target tumor cells while minimizing damage to healthy tissues. However, a comprehensive understanding of their mechanism of action remains incomplete. Key questions and even controversies persist, such as the role of autophagy and the impact of karyotipic complexity on TTFields sensitivity.

Dissecting the molecular effects of TTFields could reveal how this therapy differentially interacts with various cellular states. While it is well established that TTFields primarily affect dividing cells through antimitotic effects, how other proposed mechanisms operate in quiescent or slowly proliferating cells remains poorly understood. In particular, omics analyses of models enriched in non-proliferative or quiescent cell populations could provide valuable insights into the broader mechanisms of TTFields sensitivity. Recent findings have demonstrated that TTFields can affect bone marrow-derived macrophages – cells with limited proliferative potential – by promoting their pro-inflammatory polarization [82,83]. The impact of TTFields on the TME is an important area that warrants further attention and research, especially regarding their effects on T cells, dendritic cells, macrophages, and stromal cells, as well as the molecular factors involved in the mechanisms driving TTFields-induced ICD. Moreover, their potential to induce senescence in cancer cells and the implication of this senescence for tumor progression should be further clarified.

Addressing these issues requires robust preclinical models. Patient-derived organoids and syngeneic tumor models in immunocompetent mice could be particularly valuable in this context. Multi-omics profiling of these models, combined with imaging mass cytometry and advanced bioinformatics analysis, may help uncover the underlying complex mechanisms.

Despite increasing understanding, the wide range of cellular effects induced by TTFields makes it challenging – if not impossible – to identify reliable biomarkers or signatures for patient stratification, which would enable the selection of those most likely to benefit from the therapy. This explains why previous attempts to find parameters capable of predicting responders versus non-responders have not yielded satisfactory results. This challenge is not unique to TTFields; similar issues arise with radiotherapy and broad-spectrum anticancer drugs. For instance, despite extensive research, prognostic biomarkers for many natural products used in chemotherapy remain unidentified. Potentially, targeted translational clinical studies including molecular analyses of tumor tissues or liquid biopsies, combined with cutting-edge tools, such as single-cell RNA sequencing and machine learning algorithms for data analysis and interpretation, may help bridge this gap and identify predictive factors valuable for patient stratification.

Another crucial factor likely influencing treatment outcomes is tumor localization. Tumors situated in anatomical regions accessible to higher TTFields intensity are likely to respond better, given the localized nature of the therapy. Data from patients with GBM enrolled in the EF-14 trial showed significantly improved survival when the average local minimum field intensity was ≥1.06 V/cm RSM compared to <1.06 V/cm RSM (24.3 months versus 21.6 months, respectively). These findings highlight the importance of personalized device placement and dynamic array adjustment to maximize field coverage and intensity [84,85].

Improving the therapeutic efficacy of TTFields also involves better understanding their interaction with chemotherapeutic agents. Key open questions include identifying the most effective co-treatment strategies, determining the optimal timing for initiating treatment, assessing whether the intensity or frequency of TTFields should be adjusted when used with drugs, and exploring if drug dosages can be reduced when administered alongside TTFields.

Notably, TTFields have been shown to inhibit several DNA damage repair pathways [32], suggesting potential synthetic lethality when used with DNA damage repair inhibitors. However, additional mechanisms are likely involved. TTFields may alter drug pharmacokinetics, affecting their distribution within tumor cells and tissues [86]. Furthermore, evidences suggests that TTFields can increase cell membrane permeability, facilitating the uptake of exogenous compounds [[87], [88], [89]], and may even reversibly open the blood-brain barrier to enhance drug delivery [90]. For certain tumor types, this effect appears more pronounced when cells are treated at frequencies different from those used in standard therapy [91]. Understanding the kinetics of these phenomena is crucial, as TTFields-induced changes in cell membrane permeability and cytoskeletal disruption are transient and reversible. This suggests that prolonged exposure to TTFields is necessary to maximize their therapeutic impact during co-treatments.

As understanding of these phenomena deepens, more rational and personalized clinical applications can be developed. So far, clinical trials have primarily evaluated concurrent TTFields and standard-of-care treatments, or TTFields alone as maintenance therapy. However, preclinical data suggest that alternative treatment schedules could be considered. For instance, initial exposure to TTFields may enhance membrane permeability and/or reverse immunosuppression in the TME, potentially increasing the efficacy of the subsequent co-administration of TTFields with anticancer drugs or immune checkpoint inhibitors.

Although TTFields therapy is applied locally, improved outcomes have been observed in patients with metastatic NSCLC receiving concurrent immunotherapy, suggesting a potential abscopal effect [6]. Robust preclinical data and preliminary analyses of patient biopsies indicate that TTFields may elicit a systemic immune response against cancer. We believe that this immunomodulatory effect may be among the most clinically relevant aspects of TTFields therapy, and it could be harnessed to develop more effective anticancer strategies. Despite the significant advances of immunotherapy in oncology, many tumors remain resistant to immune checkpoint inhibitors. TTFields may offer a novel strategy to overcome both local and systemic immunosuppression, thereby enhancing the efficacy of immunotherapeutic approach.

## Funding

This work was supported by the 2021 AACR-Novocure Tumor Treating Fields Research Grant [grant number 21–60–62-DINC]; Fondazione Buzzi Unicem Onlus.

## Data availability

Data are available in the open repository Zenodo in the “IRCCS Humanitas Research Hospital & Humanitas University” community (DOI: 10.5281/zenodo.17099724).

## CRediT authorship contribution statement

**Ilaria Fuso Nerini:** Writing – original draft, Conceptualization. **Rosy Amodeo:** Writing – review & editing, Visualization, Investigation. **Maurizio D’Incalci:** Writing – review & editing, Funding acquisition, Conceptualization. **Monica Lupi:** Writing – original draft, Conceptualization.

## Declaration of competing interest

The authors declare that they have no known competing financial interests or personal relationships that could have appeared to influence the work reported in this paper.

## Appendix Supplementary materials

Supplementary Table: List of the published studies considered for the preparation of Fig. 2 and 3. Image, application 1
