# Supplementary material for: Selective antitumor activity of Tumor Treating Fields (TTFields) involving molecular factors in cancer cells and tumor microenvironment
Source: Transl Oncol. 2025 Sep 30;62:102556. doi: 10.1016/j.tranon.2025.102556 (PMC12517075; doi:10.1016/j.tranon.2025.102556)
Supplement: Supplementary file 1 — Supplementary Table: List of the published studies considered for the preparation of Fig. 2 and 3. [file mmc1.docx]

**Supplementary Table: List of the published studies considered for the preparation of Figure 2 and Figure 3**

| **Tumor** | **Cell Lines** | **TTFields intensity (V/cm RSM)** | **%Growth inhibition at 72h** | **Basal doubling time (h)** | **Ref.** |
| --- | --- | --- | --- | --- | --- |
| Human non-small cell lung cancer | NCI-H157 | 1.55 | 72 | 36 | [1] |
| Human non-small cell lung cancer | NCI-H4006 | 1.55 | 69 | 34 |  |
| Human non-small cell lung cancer | NCI-H1299 | 1.55 | 32 | 20 |  |
| Human non-small cell lung cancer | NCI-H1650 | 1.55 | 21 | 26 |  |
| Human lung carcinoma | A549 | 1.55 | 48 | 22 |  |
| Chinese hamster ovarian cancer | AA8 | 1.75 | 25 | 12 | [2] |
| Human breast cancer | MCF-7 | 1.75 | 40 | 29.3 |  |
| Human breast cancer | MDA-MB-231 | 1.75 | 50 | 29.1 |  |
| Human ovarian cancer | A2780 | 1.55 | 25 | 18.7 | [3] |
| Human ovarian cancer | OVCAR3 | 1.55 | 25 | 34.7 |  |
| Human ovarian cancer | CAOV3 | 1.55 | 15 | 51 |  |
| Human ovarian cancer | A2780 | 1.7 | 70 | 18.7 | [4] |
| Rat glioma cells | F-98 | 1.7 | 75 | 20 |  |
| Human ovarian cancer | OVCAR3 | 1.7 | 60 | 34.7 |  |
| Human glioblastoma | U-87 MG | 1.7 | 60 | 34 |  |
| Human colorectal cancer | HCT116 | 1.5 | 60 | 17.4 | [5] |
| Human colorectal cancer | SW480 | 1.5 | 40 | 24 |  |
| Human breast cancer | MDA-MB-231 | 0.63 | <10 | 29.1 | [6] |
|  | MDA-MB-231 | 1.25 | 20 | 29.1 |  |
|  | MDA-MB-231 | 1.48 | 33 | 29.1 |  |
|  | MDA-MB-231 | 1.75 | 45 | 29.1 |  |
| Human glioblastoma | U-118 MG | 1.75 | 30 | 18.5 |  |
| Human pancreatic adenocarcinoma | AsPC-1 | 2.9 | 35 | 54 | [7] |
| Human non-small cell lung cancer | NCI-H1299 | 1.75 | 50 | 20 | [8] |
| Human non-small cell lung cancer | NCI-H520 | 1.75 | 45 | 60 |  |
| Human non-small cell lung cancer | HCC827 | 1.75 | 40 | 48 |  |
| Human lung carcinoma | A549 | 1.75 | 75 | 23.8 |  |
| Murine Lewis lung carcinoma | LLC-1 | 1.75 | 50 | 21 |  |
| Murine lung squamous cell carcinoma | KLN205 | 1.75 | 60 | 31 |  |
| Human ovarian cancer | A2780 | 1.75 | 60 | 18.7 | [9] |
| Human lung carcinoma | A549 | 1.75 | 45 | 22 |  |
| Human pancreatic adenocarcinoma | AsPC-1 | 1.75 | 20 | 54 |  |
| Human breast cancer | MCF-7 | 1.75 | 30 | 29.3 |  |
| Human breast cancer | MDA-MB-231 | 1.75 | 40 | 29.1 |  |
| Human biphasic pleural mesothelioma | MSTO-211H | 1.75 | 55 | 26.4 |  |
| Human non-small cell lung cancer | NCI-H1299 | 1.75 | 60 | 23.1 |  |
| Human epithelioid pleural mesothelioma | NCI-H2052 | 1.75 | 70 | 18.9 |  |
| Human glioblastoma | U-118 MG | 1.75 | 50 | 18.5 |  |
| Human glioblastoma | U-87 MG | 1.75 | 35 | 34 |  |
| Human glioblastoma | LN-229 | 2 | 35* | 30 | [10] |
| Human glioblastoma | U-118 MG | 1.75 | 55 | 18.5 | [11] |
| Human glioblastoma | LN-18 | 1 | 80 | 33 |  |
| Human glioblastoma | U-87 MG | 1.7 | 60 | 48 | [12] |
| Human glioblastoma | U-138 MG | 1.7 | 80 | 47 |  |
| Human glioblastoma | U-343 MG | 1.7 | 70 | 45 |  |
| Human glioblastoma | GaMG | 1.7 | 65 | 32 |  |
| Human pancreatic adenocarcinoma | BxPC-3 | 0.7 | 50* | 40 | [13] |
| Human glioblastoma | U251-MG | 1.41 | >90 | 22 | [14] |
| Rat glioma cells | BT4Ca | 1 | 27 | 14 | [15] |
| Rat glioma cells | C6 | 1 | 30 | 10 |  |
| Rat glioma cells | F-98 | 1 | 20 | 23 |  |
| Rat glioma cells | RG-2 | 1 | 15 | 11 |  |
| Human non-small cell lung cancer | NCI-H157 | 1.55 | 70 | 36 | [16] |
| Human non-small cell lung cancer | NCI-H4006 | 1.55 | 50 | 34 |  |
| Human non-small cell lung cancer | NCI-H1299 | 1.55 | 30 | 20 |  |
| Human lung carcinoma | A549 | 1.55 | 30 | 22 |  |
| Murine Lewis lung carcinoma | LLC-1 | 1.75 | 60 | 21 | [17] |
| Murine colon carcinoma | CT-26 | 1.75 | 50 | 22 |  |
| Human glioblastoma | U-373 MG | 0.9 | 30 | 40 | [18] |
|  | U-373 MG | 1.2 | 40 | 40 |  |
|  | U-373 MG | 1.5 | 65 | 40 |  |
| Human glioblastoma | U-87 MG | 0.9 | 25 | 34 |  |
|  | U-87 MG | 1.2 | 35 | 34 |  |
|  | U-87 MG | 1.5 | 75 | 34 |  |
| Human adult keratinocytes | HaCaT | 0.9 | <1 | 28 |  |
|  | HaCaT | 1.2 | 20 | 28 |  |
|  | HaCaT | 1.5 | 65 | 28 |  |
| Human biphasic pleural mesothelioma | MSTO-211H | 0.7 | 20 | 17.3 | [19] |
|  | MSTO-211H | 1 | 50 | 17.3 |  |
|  | MSTO-211H | 1.5 | 70 | 17.3 |  |
| Human epithelioid pleural mesothelioma | NCI-H2052 | 0.7 | 20 | 35.2 |  |
|  | NCI-H2052 | 1 | 30 | 35.2 |  |
|  | NCI-H2052 | 1.5 | 45 | 35.2 |  |
| Human breast ductal carcinoma | JIMT-1 | 0.9 | 60 | 35 | [20] |
| Human pancreatic adenocarcinoma | BxPC-3 | 0.7 | <1 | 40 | [21] |
| Human pancreatic adenocarcinoma | AsPC-1 | 0.7 | <1 | 54 |  |
| Human non-malignant pancreatic cells | CRL-4023 | 0.7 | <1 | 26 |  |
| Human glioblastoma | KNS-42 | 1.75 | 61 | 48 | [22] |
| Human glioblastoma | SF188 | 1.75 | 50 | 26 |  |
| Human glioblastoma | GIN-31 | 1.75 | 40 | 48 |  |
| Human medulloblastoma | DAOY | 1.75 | 30 | 33 |  |
| Human medulloblastoma | UW228-3 | 1.75 | 47 | 29 |  |
| Human hepatocellular carcinoma | HepG2 | 1 | 60 | 48 | [23] |
| Human hepatocellular carcinoma | Huh-7D12 | 1.7 | 63.5 | 32 |  |
| Human lung carcinoma | A549 | 1 | 40 | 22 | [24] |
| Human non-small cell lung cancer | NCI-H460 | 1 | 50 | 17.8 |  |
| Human sarcomatoid pleural mesothelioma | CD60 | 1.12 | 28 | 44.4 | [25] |
| Human sarcomatoid pleural mesothelioma | CD432 | 1.12 | 43 | 43.6 |  |
| Human epithelioid pleural mesothelioma | CD473 | 1.12 | 65 | 42.3 |  |
| Human epithelioid pleural mesothelioma | CD484 | 1.12 | 58 | 45.9 |  |
| Human biphasic pleural mesothelioma | CD487 | 1.12 | 47 | 45.6 |  |
| Human biphasic pleural mesothelioma | CD491 | 1.12 | 24 | 111.7 |  |
| Human glioblastoma | DBTRG-05MG | 1.5 | 45 | 50 | [26] |
| Human glioblastoma | U-87 MG | 1.7 | 58 | 34 | [27] |
| Human head and neck squamous cell carcinoma | Cal27 | 1.48 | 50 | 35 | [28] |
| Human head and neck squamous cell carcinoma | FaDu | 1.48 | 25 | 40 |  |
| Human glioblastoma | U-87 MG | 1.6 | 49 | 34 | [29] |
| Human breast cancer | MDA-MB-231 | 1.5 | 75 | 29.1 | [30] |
| Human breast ductal carcinoma | HCC38 | 1.5 | 30 | 51 |  |
|  | HCC38 | 3 | 40 | 51 |  |
| Human breast epithelial cells | MCF-12 | 1.5 | 10 | 35.4 |  |
| Human glioblastoma | U-87 MG | 0.83 | 45 | 34 | [31] |
| Human glioblastoma | LN-229 | 0.83 | 30 | 30 |  |
| Human glioblastoma | U-118 MG | 0.83 | 45 | 18.5 |  |
| Human glioblastoma | LN-18 | 0.83 | 30 | 33 |  |
| Human glioblastoma | U251-MG (P) | 1 | 75 | 22 | [32] |
|  | U251-MG (T) | 1 | 60 | 22 |  |
| Human glioblastoma | U-373 MG | 1 | 40 | 40 | [33] |
| Human glioblastoma | U-87 MG | 1 | 10 | 34 |  |
| Human biphasic pleural mesothelioma | MSTO-211H | 1 | <5 | 17.3 | [34] |
| Human sarcomatoid pleural mesothelioma | VAMT-1 | 1 | <10 | 24 |  |
| Human glioblastoma | U-373 MG | 0.9 | 20 | 40 | [35] |
| Human glioblastoma | U-87 MG | 0.9 | 34 | 34 |  |
| Human glioblastoma | U-373 MG | 0.9 | 30 | 40 | [36] |
|  | U-373 MG | 1.2 | 35 | 40 |  |
| Rat normal small intestine cells | IEC6 | 0.9 | 22 | 20 |  |
|  | IEC6 | 1.2 | 27 | 20 |  |
| Human melanoma | A375SM | 0.9 | 55 | 24 | [37] |
|  | A375SM | 1.2 | 60 | 24 |  |
|  | A375SM | 1.5 | 65 | 24 |  |
| Human skin normal cells | CCD-986sk | 0.9 | <10 | 40 |  |
|  | CCD-986sk | 1.2 | <10 | 40 |  |
|  | CCD-986sk | 1.5 | <10 | 40 |  |
| Mouse melanoma | B16F10 | 0.9 | 43 | 20.1 |  |
|  | B16F10 | 1.2 | 48 | 20.1 |  |
|  | B16F10 | 1.5 | 52 | 20.1 |  |
| Mouse embryo cells | NIH3T3 | 0.9 | 25 | 28 |  |
|  | NIH3T3 | 1.2 | 30 | 28 |  |
|  | NIH3T3 | 1.5 | 40 | 28 |  |
| Human breast cancer | MDA-MB-231 | 1.1 | 50 | 29.1 | [38] |
| Human lung carcinoma | A549 | 1.1 | 64 | 22 |  |
| Human pancreatic adenocarcinoma | HPAF-II | 1.1 | 40 | 40 | [39] |
| Human pancreatic adenocarcinoma | Mia-Paca II | 1.1 | 40 | 40 |  |

* %Growth inhibition calculated after 48h of treatment.

**References**

[1] N. K. Karanam, K. Srinivasan, L. Ding, B. Sishc, D. Saha, and M. D. Story, Tumor-treating fields elicit a conditional vulnerability to ionizing radiation via the downregulation of BRCA1 signaling and reduced DNA double-strand break repair capacity in non-small cell lung cancer cell lines, Cell Death Dis 8 (2017) e2711, <https://doi.org/>10.1038/cddis.2017.136.

[2] R. S. Schneiderman, E. Shmueli, E. D. Kirson, and Y. Palti, TTFields alone and in combination with chemotherapeutic agents effectively reduce the viability of MDR cell sub-lines that over-express ABC transporters, BMC Cancer 10 (2010) 229, <https://doi.org/>10.1186/1471-2407-10-229.

[3] T. Voloshin, M. Munster, R. Blatt, A. Shteingauz, P. C. Roberts, E. M. Schmelz, et al., Alternating electric fields (TTFields) in combination with paclitaxel are therapeutically effective against ovarian cancer cells in vitro and in vivo, Int J Cancer 139 (2016) 2850-2858, <https://doi.org/>10.1002/ijc.30406.

[4] Y. Porat, M. Giladi, R. S. Schneiderman, R. Blat, A. Shteingauz, E. Zeevi, et al., Determining the optimal inhibitory frequency for cancerous cells using tumor treating fields (TTFields), J Vis Exp (2017)<https://doi.org/>10.3791/55820.

[5] Y. Lee, J. Cho, S. Sai, J. Y. Oh, J. Park, S. J. Oh, et al., 5-fluorouracil as a tumor-treating field-sensitizer in colon cancer therapy, Cancers (Basel) 11 (2019) 1999, <https://doi.org/>10.3390/cancers11121999.

[6] E. D. Kirson, R. S. Schneiderman, V. Dbalý, F. Tovarys, J. Vymazal, A. Itzhaki, et al., Chemotherapeutic treatment efficacy and sensitivity are increased by adjuvant alternating electric fields (TTFields), BMC Med Phys 9 (2009) 1, <https://doi.org/>10.1186/1756-6649-9-1.

[7] M. Giladi, R. S. Schneiderman, Y. Porat, M. Munster, A. Itzhaki, D. Mordechovich, et al., Mitotic disruption and reduced clonogenicity of pancreatic cancer cells in vitro and in vivo by tumor treating fields, Pancreatology 14 (2014) 54-63, <https://doi.org/>10.1016/j.pan.2013.11.009.

[8] M. Giladi, U. Weinberg, R. S. Schneiderman, Y. Porat, M. Munster, T. Voloshin, et al., Alternating electric fields (tumor-treating fields therapy) can improve chemotherapy treatment efficacy in non-small cell lung cancer both in vitro and in vivo, Semin Oncol 41 Suppl 6 (2014) 35, <https://doi.org/>10.1053/j.seminoncol.2014.09.006.

[9] M. Giladi, R. S. Schneiderman, T. Voloshin, Y. Porat, M. Munster, R. Blat, et al., Mitotic spindle disruption by alternating electric fields leads to improper chromosome segregation and mitotic catastrophe in cancer cells, Sci Rep 5 (2015) 18046, <https://doi.org/>10.1038/srep18046.

[10] M. Silginer, M. Weller, R. Stupp, and P. Roth, Biological activity of tumor-treating fields in preclinical glioma models, Cell Death Dis 8 (2017) e2753, <https://doi.org/>10.1038/cddis.2017.171.

[11] M. Giladi, M. Munster, R. S. Schneiderman, T. Voloshin, Y. Porat, R. Blat, et al., Tumor treating fields (TTFields) delay DNA damage repair following radiation treatment of glioma cells, Radiat Oncol 12 (2017) 206, <https://doi.org/>10.1186/s13014-017-0941-6.

[12] A. F. Kessler, G. E. Frömbling, F. Gross, M. Hahn, W. Dzokou, R. Ernestus, et al., Effects of tumor treating fields (TTFields) on glioblastoma cells are augmented by mitotic checkpoint inhibition, Cell Death Discov 4 (2018) 12, <https://doi.org/>10.1038/s41420-018-0079-9.

[13] T. Pfeifer, L. Bai, J. Gladkich, W. Gross, L. Liu, I. Herr, et al., Therapy of pancreatic cancer with alternating electric fields: Limitations of the method, Bioelectrochemistry 141 (2021) 107881, <https://doi.org/>10.1016/j.bioelechem.2021.107881.

[14] B. Linder, A. Schiesl, M. Voss, F. Rödel, S. Hehlgans, Ö Güllülü, et al., Dexamethasone treatment limits efficacy of radiation, but does not interfere with glioma cell death induced by tumor treating fields, Front Oncol 11 (2021) 715031, <https://doi.org/>10.3389/fonc.2021.715031.

[15] L. Berkelmann, A. Bader, S. Meshksar, A. Dierks, G. Hatipoglu Majernik, J. K. Krauss, et al., Tumour-treating fields (TTFields): Investigations on the mechanism of action by electromagnetic exposure of cells in telophase/cytokinesis, Sci Rep 9 (2019) 7362, <https://doi.org/>10.1038/s41598-019-43621-9.

[16] N. K. Karanam, L. Ding, A. Aroumougame, and M. D. Story, Tumor treating fields cause replication stress and interfere with DNA replication fork maintenance: Implications for cancer therapy, Transl Res 217 (2020) 33-46, <https://doi.org/>10.1016/j.trsl.2019.10.003.

[17] T. Voloshin, N. Kaynan, S. Davidi, Y. Porat, A. Shteingauz, R. S. Schneiderman, et al., Tumor-treating fields (TTFields) induce immunogenic cell death resulting in enhanced antitumor efficacy when combined with anti-PD-1 therapy, Cancer Immunol Immunother 69 (2020) 1191-1204, <https://doi.org/>10.1007/s00262-020-02534-7.

[18] H. Jeong, Y. Jo, M. Yoon, and S. Hong, Thymidine decreases the DNA damage and apoptosis caused by tumor-treating fields in cancer cell lines, Genes Genomics 43 (2021) 995-1001, <https://doi.org/>10.1007/s13258-021-01105-z.

[19] H. Mumblat, A. Martinez-Conde, O. Braten, M. Munster, E. Dor-On, R. S. Schneiderman, et al., Tumor treating fields (TTFields) downregulate the fanconi anemia-BRCA pathway and increase the efficacy of chemotherapy in malignant pleural mesothelioma preclinical models, Lung Cancer 160 (2021) 99-110, <https://doi.org/>10.1016/j.lungcan.2021.08.011.

[20] J. S. Kim, J. M. Cho, H. Kim, Y. K. Jeong, J. Kim, and E. H. Kim, Tumor treating fields can effectively overcome trastuzumab resistant breast cancer multiplication, Am J Cancer Res 11 (2021) 3935-3945, .

[21] L. Bai, T. Pfeifer, W. Gross, C. De La Torre, S. Zhao, L. Liu, et al., Establishment of tumor treating fields combined with mild hyperthermia as novel supporting therapy for pancreatic cancer, Front Oncol 11 (2021) 738801, <https://doi.org/>10.3389/fonc.2021.738801.

[22] J. Branter, M. Estevez-Cebrero, M. Diksin, M. Griffin, M. Castellanos-Uribe, S. May, et al., Genome-wide expression and anti-proliferative effects of electric field therapy on pediatric and adult brain tumors, Int J Mol Sci 23 (2022) 1982, <https://doi.org/>10.3390/ijms23041982.

[23] S. Davidi, S. Jacobovitch, A. Shteingauz, A. Martinez-Conde, O. Braten, C. Tempel-Brami, et al., Tumor treating fields (TTFields) concomitant with sorafenib inhibit hepatocellular carcinoma in vitro and in vivo, Cancers (Basel) 14 (2022) 2959, <https://doi.org/>10.3390/cancers14122959.

[24] W. S. Lee and E. H. Kim, Combination therapy of doxorubicin with TTFields and radiation: Newer approaches to combat lung cancer, Am J Cancer Res 12 (2022) 2673-2685, .

[25] L. Mannarino, F. Mirimao, N. Panini, L. Paracchini, S. Marchini, L. Beltrame, et al., Tumor treating fields affect mesothelioma cell proliferation by exerting histotype-dependent cell cycle checkpoint activations and transcriptional modulations, Cell Death Dis 13 (2022) 612, <https://doi.org/>10.1038/s41419-022-05073-4.

[26] S. Xu, C. Luo, D. Chen, L. Tang, L. Chen, and Z. Liu, Whole transcriptome and proteome analyses identify potential targets and mechanisms underlying tumor treating fields against glioblastoma, Cell Death Dis 13 (2022) 721, <https://doi.org/>10.1038/s41419-022-05127-7.

[27] V. Nickl, E. Schulz, E. Salvador, L. Trautmann, L. Diener, A. F. Kessler, et al., Glioblastoma-derived three-dimensional ex vivo models to evaluate effects and efficacy of tumor treating fields (TTFields), Cancers (Basel) 14 (2022) 5177, <https://doi.org/>10.3390/cancers14215177.

[28] S. Regnery, H. Franke, T. Held, T. Trinh, A. Naveh, Y. Abraham, et al., Tumor treating fields as novel combination partner in the multimodal treatment of head and neck cancer, Head Neck 45 (2023) 838-848, <https://doi.org/>10.1002/hed.27298.

[29] D. Krex, P. Bartmann, D. Lachmann, A. Hagstotz, W. Jugel, R. S. Schneiderman, et al., Aurora B kinase inhibition by AZD1152 concomitant with tumor treating fields is effective in the treatment of cultures from primary and recurrent glioblastomas, Int J Mol Sci 24 (2023) 5016, <https://doi.org/>10.3390/ijms24055016.

[30] A. R. Smothers, J. R. Henderson, J. J. O'Connell, J. M. Stenbeck, D. Dean, and B. W. Booth, Optimization of tumor-treating field therapy for triple-negative breast cancer cells in vitro via frequency modulation, Cancer Cell Int 23 (2023) 110, <https://doi.org/>10.1186/s12935-023-02959-x.

[31] H. Fishman, R. Monin, E. Dor-On, A. Kinzel, A. Haber, M. Giladi, et al., Tumor treating fields (TTFields) increase the effectiveness of temozolomide and lomustine in glioblastoma cell lines, J Neurooncol 163 (2023) 83-94, <https://doi.org/>10.1007/s11060-023-04308-4.

[32] A. B. Jones, T. L. Schanel, M. R. Rigsby, C. E. Griguer, B. C. McFarland, J. C. Anderson, et al., Tumor treating fields alter the kinomic landscape in glioblastoma revealing therapeutic vulnerabilities, Cells 12 (2023) 2171, <https://doi.org/>10.3390/cells12172171.

[33] E. H. Kim, Tumor treating fields induced senescence on glioblastoma, Am J Cancer Res 13 (2023) 5626-5640, .

[34] A. Sarkari, S. Korenfeld, K. Deniz, K. Ladner, P. Wong, S. Padmanabhan, et al., Treatment with tumor-treating fields (TTFields) suppresses intercellular tunneling nanotube formation in vitro and upregulates immuno-oncologic biomarkers in vivo in malignant mesothelioma, Elife 12 (2023) e85383, <https://doi.org/>10.7554/eLife.85383.

[35] E. H. Kim, H. S. Song, S. H. Yoo, and M. Yoon, Tumor treating fields inhibit glioblastoma cell migration, invasion and angiogenesis, Oncotarget 7 (2016) 65125-65136, <https://doi.org/>10.18632/oncotarget.11372.

[36] Y. Jo, J. Sung, H. Jeong, S. Hong, Y. K. Jeong, E. H. Kim, et al., Effectiveness of a fractionated therapy scheme in tumor treating fields therapy, Technol Cancer Res Treat 18 (2019) 1533033819845008, <https://doi.org/>10.1177/1533033819845008.

[37] Y. Jo, S. Hwang, Y. B. Jin, J. Sung, Y. K. Jeong, J. H. Baek, et al., Selective toxicity of tumor treating fields to melanoma: An in vitro and in vivo study, Cell Death Discov 4 (2018) 46, <https://doi.org/>10.1038/s41420-018-0106-x.

[38] A. Pavesi, G. Adriani, A. Tay, M. E. Warkiani, W. H. Yeap, S. C. Wong, et al., Engineering a 3D microfluidic culture platform for tumor-treating field application, Sci Rep 6 (2016) 26584, <https://doi.org/>10.1038/srep26584.

[39] Y. Jo, E. Lee, G. Oh, Y. Gi, and M. Yoon, Synergistic effect of TTF and 5-FU combination treatment on pancreatic cancer cells, Am J Cancer Res 13 (2023) 4734-4741, .
